# Chromosome-level genome assembly of *Oncomelania hupensis:* the intermediate snail host of *Schistosoma japonicum*

**DOI:** 10.1186/s40249-024-01187-3

**Published:** 2024-02-27

**Authors:** Qin Liu, Lei Duan, Yun-Hai Guo, Li-Min Yang, Yi Zhang, Shi-Zhu Li, Shan Lv, Wei Hu, Nan-Sheng Chen, Xiao-Nong Zhou

**Affiliations:** 1 National Institute of Parasitic Diseases at Chinese Center for Disease Control and Prevention (Chinese Center for Tropical Diseases Research); NHC Key Laboratory of Parasite and Vector Biology; WHO Collaborating Centre for Tropical Diseases; National Center for International Research on Tropical Diseases; National Key Laboratory of Intelligent Tracking and Forecasting for Infectious Diseases, Shanghai, 200025 People’s Republic of China; 2 School of Global Health, Chinese Center for Tropical Diseases Research, Shanghai Jiao Tong University School of Medicine, Shanghai, 200025 People’s Republic of China; 3 School of Life Science, Fudan University, Shanghai, 200438 People’s Republic of China; 4 CAS Key Laboratory of Marine Ecology and Environmental Sciences, Institute of Oceanology, Chinese Academy of Sciences, Qingdao, Shandong 266071 People’s Republic of China

**Keywords:** Schistosomiasis, *Schistosoma japonicum*, *Oncomelania hupensis*, Chromosome-level genome

## Abstract

**Background:**

*Schistosoma japonicum* is a parasitic flatworm that causes human schistosomiasis, which is a significant cause of morbidity in China, the Philippines and Indonesia. *Oncomelania hupensis* (Gastropoda: Pomatiopsidae) is the unique intermediate host of *S. japonicum*. A complete genome sequence of *O. hupensis* will enable the fundamental understanding of snail biology as well as its co-evolution with the *S. japonicum* parasite. Assembling a high-quality reference genome of *O. hupehensis* will provide data for further research on the snail biology and controlling the spread of *S. japonicum.*

**Methods:**

The draft genome was de novo assembly using the long-read sequencing technology (PacBio Sequel II) and corrected with Illumina sequencing data. Then, using Hi-C sequencing data, the genome was assembled at the chromosomal level. CAFE was used to do analysis of contraction and expansion of the gene family and CodeML module in PAML was used for positive selection analysis in protein coding sequences.

**Results:**

A total length of 1.46 Gb high-quality *O. hupensis* genome with 17 unique full-length chromosomes (2n = 34) of the individual including a contig N50 of 1.35 Mb and a scaffold N50 of 75.08 Mb. Additionally, 95.03% of these contig sequences were anchored in 17 chromosomes. After scanning the assembled genome, a total of 30,604 protein-coding genes were predicted. Among them, 86.67% were functionally annotated. Further phylogenetic analysis revealed that *O. hupensis* was separated from a common ancestor of *Pomacea canaliculata* and *Bellamya purificata* approximately 170 million years ago. Comparing the genome of *O. hupensis* with its most recent common ancestor, it showed 266 significantly expanded and 58 significantly contracted gene families (*P* < 0.05). Functional enrichment of the expanded gene families indicated that they were mainly involved with intracellular, DNA-mediated transposition, DNA integration and transposase activity.

**Conclusions:**

Integrated use of multiple sequencing technologies, we have successfully constructed the genome at the chromosomal-level of *O. hupensis*. These data will not only provide the compressive genomic information, but also benefit future work on population genetics of this snail as well as evolutional studies between *S. japonicum* and the snail host.

**Graphical Abstract:**

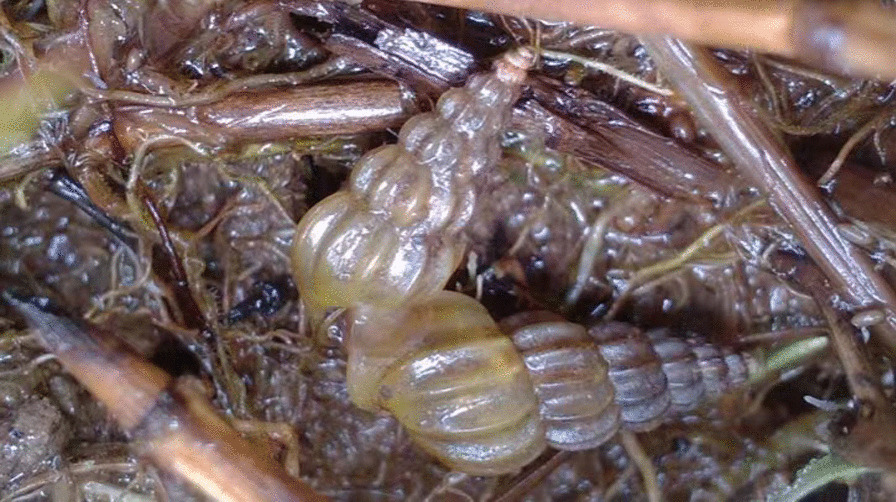

**Supplementary Information:**

The online version contains supplementary material available at 10.1186/s40249-024-01187-3.

## Background

Schistosomiasis is a zoonotic disease estimated to affect over 250 million people in the world [1]. In Southeast Asia, including China, the Philippines and Indonesia, the causative parasite of the disease, *Schistosoma japonicum*, produced major public health problems [2]. In China, a total of 452 endemic counties, including 27,434 endemic villages covering 73 million people, were once at risk for this infection, with over 30 thousand advanced schistosomiasis cases remained [3]. Over 7 decades of integrated control efforts have led to that 343 among 452 endemic counties (75.88%) now meet the standards for elimination of schistosomiasis, 106 (23.45%) for transmission interruption, and 3 (0.66%) for transmission control [3]. However, there are still reports of acute schistosomiasis cases as well as newly discovered infested snails [4].

*Oncomelania hupensis*, an amphibious snail species, is the unique intermediate host of *S. japonicum*. Without distribution of *O. hupensis,* the area would be no any transmission of schistosomiasis japonica. However, the areas inhabited with *O. hupensis* have remained at about 3.58 billion square meters since 2016 in China, and newly emerging and re-emerging snail habitats have been reported in many areas of the country [5]. The breeding sites and spreading of *O. hupensis* are the hotbed for the transmission and retransmission of schistosomiasis japonica, even after elimination. It has been reported that spreading snails which even after migrating from permissive area to non-permissive area for 13 years did not change their schistosomiasis transmission ability [6]. Together with preventive chemotherapy of people located in endemic areas, snail control therefore plays an important role in consolidating the achievements made so far in the national schistosomiasis control and elimination program in China.

To date, a variety of mollusk genomes have been analyzed and published, including those of four freshwater gastropod snails, *Pomacea canaliculata* [7], *Biomphalaria glabrata* [8], *B. pfeifferi* [9] and *Bellamya purificata* [10]. However, no genome has been reported for the Pomatiopsinae. The lack of a reference genome for this family has limited research on its evolution and biology needed for its control. Pomatiopsidae comprises two subfamilies, the Triculinae and Oncomelaniae. The former includes *Neotricula aperta* that is present in an area stretching from northern India into Southeast Asia including southern China. It is the intermediate host of *S. mekongi*, a species only found in limited areas along the Mekong River in Lao People’s Democratic Republic and Cambodia, while the latter includes *O. hupensis* that transmits *S. japonicum* in China, the Philippines and Sulawesi island of Indonesia [11]. The species of Pomatiopsidae are of great interest due to their parasitological importance, especially the Oncomelaniae, which is amphibious and only distributed in China, Japan and Southeast Asia, obviously with poor further dispersal capabilities. Although importance related to the disease transmission, those snails remain many questions regarding their biological issues. Previous surveillance from China showed that *O. hupensis* snails mainly distributed in three complicated ecologically environments, such as plain and water network region,  lake and marshland region, and mountainous and hilly region. And research results showed that in different environments, the snails had different morphology in shell and susceptibility to *S. japonicum*. Generally, the ribbed-shell snails mainly distributed in plain and water network region as well as lake and marshland region were more susceptible to *S. japonicum* than that of smooth-shell snails from mountainous and hilly region [12]. However, the mechanism of differences in morphology and susceptibility to parasites between ribbed-shell and smooth-shell snails was still unclear until now. In this study, PacBio long-read sequencing and high-throughput chromosome conformation capture (Hi-C) technology were used to assemble a high-quality chromosome-level genome of *O. hupensis*, which could provide crucial impetus to studies on origin, taxonomy, population genetics and co-evolution with *Schistosoma* spp. Genetic information from genome sequencing of Pomatiopsidae mollusk could provide an important reference to the study on the molecular mechanisms of biological control of this intermediate host snail.

## Methods

### Sample preparation

**Fig. 1:**
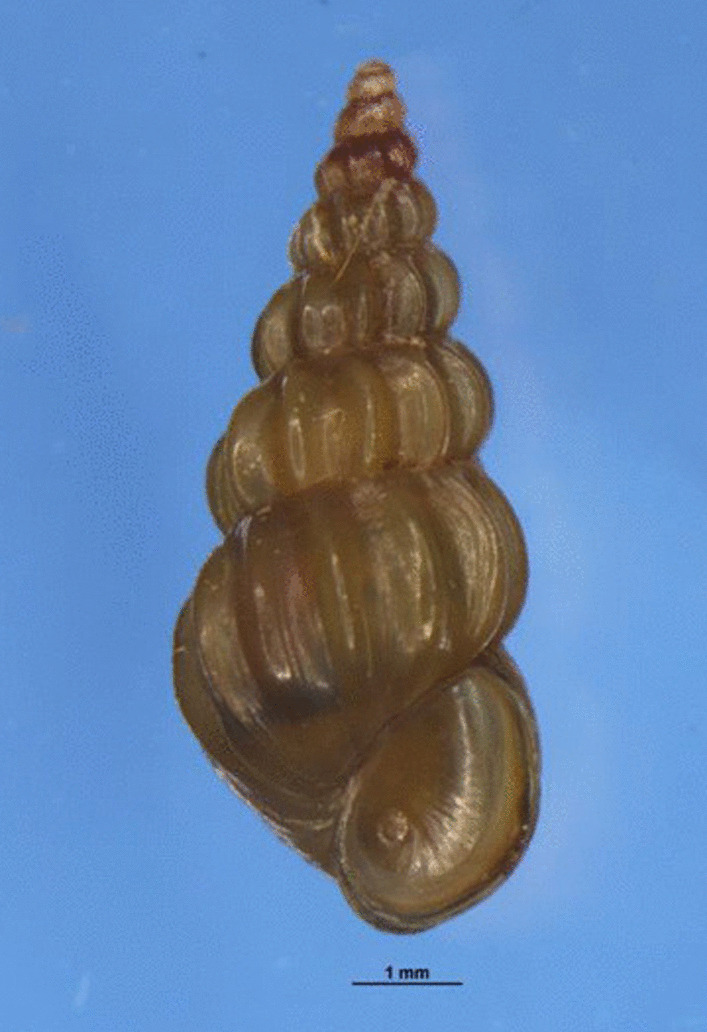
The morphology of the *O. hupensis* snail which used for genome sequencing

A second-generation, adult male *O. hupensis* offspring collected from a laboratory population breeding facility that originally from Guichi County, Anhui Province (E: 117.4477, N: 30.6581) (Fig. 1), was used for reference genome construction. The snail was dissected into abdominal foot and liver pancreas tissues, which were quickly frozen in liquid nitrogen at − 80 ℃ overnight before transfer to storage.

### Genomic DNA preparation and genome sequencing

DNA was extracted using the traditional phenol/chloroform extraction method and the library constructed for sequencing. DNA degradation and contamination was detection by 1% agarose gel. The gDNA integrity was assessed by an Agilent bioanalyzer 2100 (Agilent Technologies, Santa Clara, CA, USA) and the concentration measured by Qubit 3.0 (Invitrogen, Waltham, MA, USA). Two high-throughput sequencing platforms were used: the PacBio Sequel II (Pacific Biosciences, Menlo Park, CA, USA) and Illumina NovaSeq 6000 (Illumina Inc., San Diego, CA, USA). For PacBio SMRT sequencing, a single standard SMRTbell library with an average insert size of 20 kbp was constructed from more than 10 µg of gDNA using SMRTbell template prep kits according to the manufacturer’s protocol. Subsequent sequencing was performed on the PacBio Sequel II System at Frasergen Bioinformatics Co., Ltd. (Wuhan, China). A total of 127.92 Gb PacBio data with an average sub-read length of 8.02 kbp was produced, a 300–350 bp library constructed and the clean data, amounting to 118.78 Gb, obtained.

To improve the completeness of the assembled genome, we used a male adult *O. hupensis* with ribbed-shell from the same area for chromosome conformation capture (Hi-C) experiments. The biotinylated DNA fragments were sheared to 300–500 bp by sonication and specifically enriched with streptavidin magnetic beads for paired-end Hi-C library preparation. These Hi-C libraries were sequenced on the Illumina NovaSeq 6000, yielding 132.58 Gb of raw data (roughly 90.91 × coverage of the assembled genome).

**Fig. 2:**
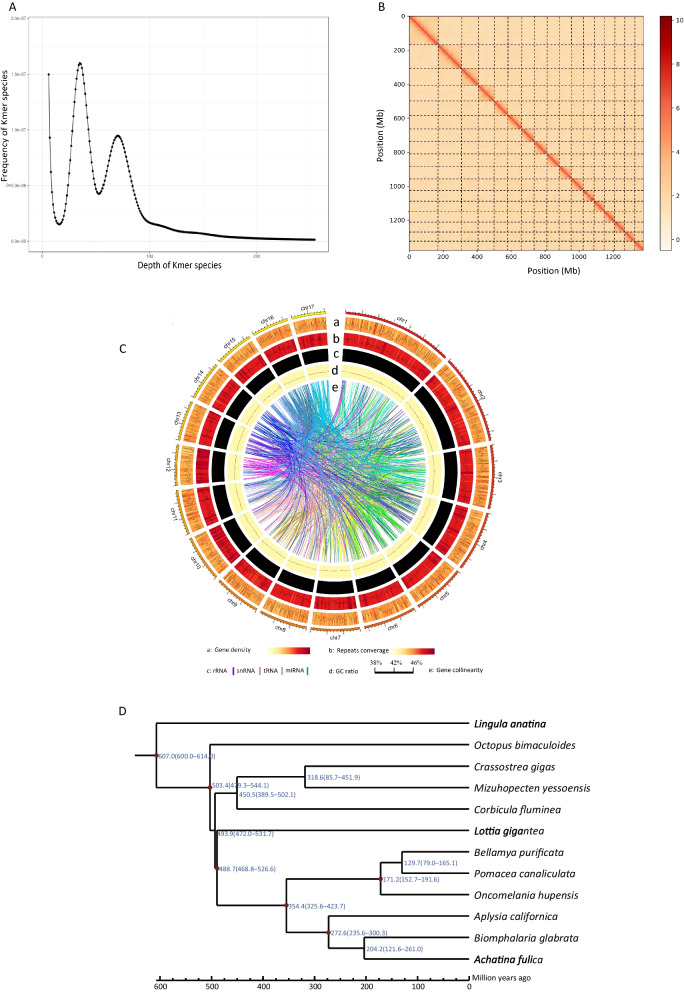

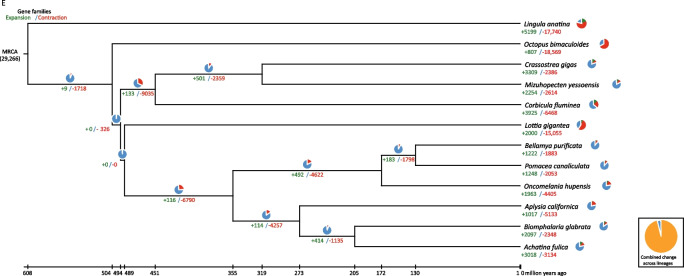
Characterization of chromosome-level genome of *O. hupensis*. **A** Frequency distribution of k-mer depth and k-mer species. **B** Hi-C interaction heatmap at a resolution of 200 kb. **C** The landscape of genome assembly and annotation of *O. hupensis*. **D** Estimates of species divergence times. **E** Number of expanded and contracted gene families in *O. hupensis*

To estimate the genome size of *O. hupensis*, we performed k-mer (k = 17) frequency distribution analysis using 105.84 Gb of clean data (Fig. 2A). In this process, 17 bp k-mers (17-mer) were extracted from the sequencing data and 17-mer frequency was calculated.

### De novo genome assembly and quality assessment

The contig-level assembly was performed with full PacBio long reads using Next Denovo (version 0.5) [13]. The consensus sequences of the assembly were further corrected with PacBio reads using GCPP (pb-assembly 0.0.8, Pacific Biosciences, San Diego, California, USA) and Illumina clean reads using Pilon (version 1.22) with three iterations in each case [14]. The contigs were corrected for order and orientation, and anchored into a candidate chromosome-length assembly with Hi-C data using Juicer (version 1.6.2) [15] and 3D-DNA pipeline (version 180419) [16]. The completeness and continuity of the *O. hupensis* assembly were then assessed with BUSCO (version 3.0, metazoa_odb9) [17]. The Illumina short reads were mapped to the *O. hupensis* assembly using the BWA-MEM module (version 0.7.17), a widely-used algorithm for genomic short read mapping [18]. Picard (version 2.19.0) was applied to mask the polymerase chain reaction (PCR) duplicates and generate the dedup.bam file. Variants (SNPs + INDELs) were called by GATK (version 1.8.0) [19].

### Transcriptome analysis

For long-read RNA sequencing (Iso-Seq), total RNA was extracted from three male and three female snails using the TRIzol extraction reagent (ThermoFisher). RNA purity was checked using the kaiaoK5500®Spectrophotometer (Kaiao, Beijing, China). RNA integrity and concentration was assessed using the RNA Nano 6000 Assay Kit of the Bioanalyzer 2100 system (Agilent Technologies, CA, USA). After mixing equal amounts of extracted RNA, Iso-Seq SMRT bell libraries were prepared and then sequenced by the PacBio Sequel II platform, producing 14.45 Gb of full-length transcriptome data. Additionally, 150 bp PE RNA-Seq libraries were constructed with a TruSeq RNA Library Preparation Kit v2 and sequenced on an Illumina NovaSeq 6000 at Novogene Co., Ltd. Fastp (version 0.19.3) was applied to remove the adaptor and low-quality reads to generate clean short reads, which were used for the construction of transcriptions.

### Repeat annotation

Repeat elements were identified with a combination of the de novo repeat library and homology-based strategies. GenomeTools suite (LTRharvest and LTRdigenst) was used to collect LTR retrotransposons with protein profile hidden Markov models (HMMs) from the Pfam database, another de novo repeat library was constructed by RepeatModeler and all three repeat libraries collected were used to search against a metazoan protein database to exclude protein-coding gene fragments [20]. Then RepeatMasker was used to discover and identify repeat elements in the *O. hupensis* genome with the combined library of the de novo repeat library and Repbase database [21, 22].

### Protein-coding genes prediction and functional annotation

Gene models were constructed with MAKER2 that incorporates the ab initio prediction, homology-based prediction and transcriptome assisted gene prediction [23]. For the homology-based prediction, we collected proteins from six sequenced and annotated mollusks, including *B. glabrata* [8], *P. canaliculata* [7], *Achatina fulita* [24], *Lottia gigantea* [25], *Aplysia californica* [26] and *Mizuhopecten yessoensis* [27], which were initially mapped onto the *O. hupensis* genome using tBlastn (version 2.2.0) to polish the BLAST hits and acquire the exact intron/exon position [28, 29]. Prediction of transcriptome transcripts based on Transcriptome RNAseq/ISO seqand HISAT2 were used for data comparison, StringTie for transcript prediction, ISOseq3 for full transcript acquisition of PacBio and TransDecoder for Coding region prediction [30, 31]. The repeat regions in the *O. hupensis* assembly were soft-masked, and with Augustus, Genscan and GeneID performed to predict protein-coding genes [32, 33]. Ultimately, MAKER2 was applied to generate consensus gene model with all these confirmatory data. The completeness of genome annotation was also measured using BUSCO (version 3) [17].

Gene functional annotations were assigned according to the best match by aligning the protein sequences to Swiss-Prot, TrEMBL and National Center for Biotechnology Information (NCBI) non-redundant (NR) databases (with a threshold of E-value 1e-5). The motifs and domains were annotated using InterProScan (version 5) [34]. GhostKOALA was applied to search the Kyoto Encyclopedia of Genes and Genomes (KEGG) database for KEGG Orthology (KO) assignments and for generating a KEGG pathway membership [35].

### Phylogenetic analysis

OrthMCL was used to cluster gene families. First, proteins from *O. hupensis* and the closely related mollusks including *P. canaliculata*, *A. fulica*, *B. glabrata*, *L. gigantea*, *A. californica*, *Lingula anatine*, *M. yessoensis*, *B. apurificata*, *Octopus bimaculoides*, *Crassostrea gigas*, *Corbicula fluminea* were all-to-all blasted by a BLASTP utility with an e-value threshold of 1e-5. Protein sequences of single-copy genes were aligned using MUSCLE [36]. The phylogenetic relationships were constructed using PhyML [37] based on the concatenated nucleotide alignment with the JTT + G + F model. The divergent times for all pairs with the phylogenetic tree were obtained by using the r8s [38] and MCMCtree programs (from PAML) [39] together with molecular clock data from the divergence time from the TimeTree database [40].

With respect gene family expansion, CAFE was used to do analysis of contraction and expansion, while the PAML CodeML module was used for positive selection analysis in protein coding sequences [41, 42].

### Data availability

All raw sequencing data generated here have been deposited in the public database NCBI Sequence Read Archive (SRA), and annotated genome assembly results have been uploaded to GenBank under the bioproject number PRJNA1033027 and JBAHVR000000000. Genome-seq for the *L. anatine* (https://www.ncbi.nlm.nih.gov/genome/?term=Lingula+anatine), *L. gigantea* (https://www.ncbi.nlm.nih.gov/genome/?term=Lottia+gigantea), *O. bimaculoides* (https://www.ncbi.nlm.nih.gov/genome/?term=Octopus+bimaculoides), *M. yessoensis* (https://www.ncbi.nlm.nih.gov/genome/?term=Mizuhopecten+yessoensis), *C. gigas* (https://www.ncbi.nlm.nih.gov/genome/?term=crassostrea+gigas%5Borgn%5D), *C. fluminea* (https://figshare.com/articles/dataset/Dissectingthe_chromosome-level_genome_of_Asian_Clam_Corbicula_fluminea_/12805886/1), *M. coruscus* (https://ftp.ncbi.nlm.nih.gov/genomes/all/GCA/011/752/425/GCA011752425_.2_MCOR1.1/), *A. californica* (https://www.ncbi.nlm.nih.gov/genome/?term=Aplysia+californica), *A. fulica* (http://gigadb.org/dataset/100647), *B. glabrata* (https://www.ncbi.nlm.nih.gov/genome/357?genome_assembly_id=2130520) and *B. purificata* (PRJNA818874) [10] were retrieved from NCBI.

## Results

### Genome sequencing, assembly and annotation

The 17-mer analysis conformed to Poisson distribution, with an estimated genome size of 1.46 Gb and heterozygous ratio of 1.69% as well as the repeat sequence proportion was 64.16% and genome GC-content was about 41.05%, respectively. A genome size of 2.69 Gb was obtained by the data assembled by NextDenovo. This was polished with GCPP and Pion, and a haplotigs purge was performed to reduce the genome size to 1.54 Gb, which was consistent with that estimated by k-mer analysis. The total number of contigs was 1512, with a contig N50 of 1.83 Mb. Using the Hi-C platform, we anchored 1376.90 Mb contig sequences to 17 super-scaffolds (chromosomes) (Fig. 2B). The final assembly yielded a high-quality genome of 1449.86 Mb, with 2178 contigs, a contig N50 of 1.35 Mb, and a scaffold N50 of 75.08 Mb (Table 1 and Additional file 1: Tables S1–S3). 91.10% of the BUSCO genes were identified in the *O. hupensis* genome and more than 88.34% of them were single-copy ones (Additional file 1: Table S4). We further evaluated this draft assembly by BWA-MEM to mapping Illumina data to the genome assembly and found the genome coverage was 99.65% and the mapping rate 99.15% (Additional file 1: Table S5).

**Table 1:** Assembly features for the *O. hupensis* genome

Assembly feature	Value
Estimated genome size	1458.42 Mb
Assembly size	1449.86 Mb
Total length of contigs	1448.99 Mb
Scaffold N50	75.08 Mb
Contig N50	1.35 Mb
GC content	40.78%
Chromosome number	17
Total length of chromosome	1377.77 Mb
Total repeat size	759.49 Mb
Gene number	30,604
Average gene length	20,007 bp

In total, according to protein-homology-based prediction methods, as well as supported by transcriptome data, 30,604 high-confidence protein-coding genes were identified and predicted with an average coding sequence (CDS) length of 1396.39 bp and an average gene length of 20,007.73 bp. Among these, 26,526 (86.67%) of the predicted protein-coding genes could be mapped with functional annotations using public databases (Fig. 2C and Additional file 1: Table S6). Through a combination of homology-based searches and de novo prediction, 759.49 Mb repetitive sequences were identified, accounting for 52.38% of the genome size. Among the repetitive sequences, DNA transposons, long interspersed nuclear elements, short interspersed nuclear elements and long terminal repeats accounted for 11.19%, 29.04%, 0.73%, and 19.09% of the genome size, respectively (Fig. 2C and Additional file 1: Tables S7, S8). We also identified 1403 non-coding RNAs (nRNAs), the prediction of which, we successfully annotated 10 microRNAs (miRNAs); 1307 transfer RNAs (tRNAs);17 ribosomal RNAs (rRNAs), and 69 small non-coding RNAs (snRNAs) with a total length of 117,495 bp and an average length of 84 bp (Additional file 1: Table S9).

### Phylogenetic analysis of *O. hupensis* with other mollusks

Comparison of the *O. hupensis* genome with that of eleven other mollusk species (*P. canaliculata*, *B. glabrata*, *A. fulica*, *L. gigantea*, *B. purificata*, *A. californica*, *L. anatine*, *M. yessoensis*, *C. gigas*, *C. fluminea*, *O. bimaculoides*) revealed a total of 4617 gene families and 1196 single-copy genes. The *O. hupensis* genome contained a total of 26,089 genes clustered into 12,366 gene families, including 707 unique families. Average gene number per family ranged from 1.27 (*A. californica*) to 2.11 (*O. hupensis*) for the twelve species (Additional file 1: Table S10).

Based on the protein sequences of the single-copy genes, we constructed a phylogenetic tree showing that *O. hupensis* was most closely related to *P. canaliculata* and *B. purificata*  that diverged from a common ancestor around 152.70–191.60 million years ago (MYA) (Fig. 2D).

### Gene family expansion, contraction and positive selection analysis

**Fig. 3:**
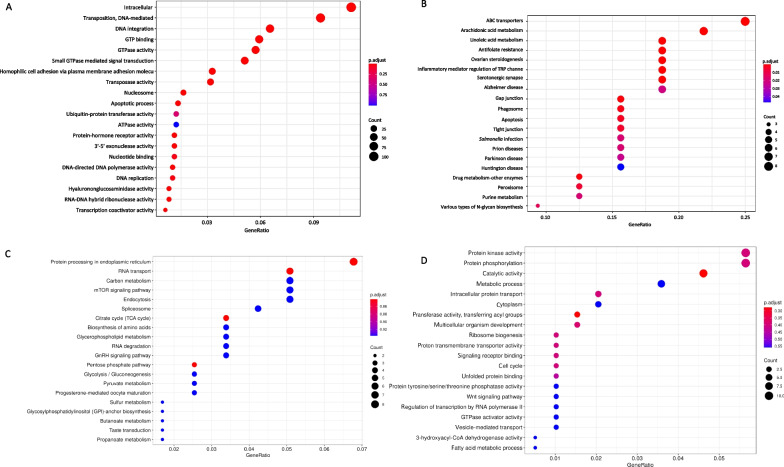
GO and KEGG analysis of gene family. **A** Significantly expanded gene family (*P* < 0.05) GO enrichment bubble plot. **B** Significantly contracted gene family (*P* < 0.05) KEGG enrichment plot. **C** KEGG enrichment analysis of positively selected genes. **D** GO enrichment analysis of positively selected genes

Gene family analysis performed with CAFE showed 266 significantly expanded gene families (*P* < 0.05), while 58 significantly contracted gene families (*P* < 0.05) were found by comparing the *O. hupensis* genome with its most recent common ancestor. Compared with *P. canaliculata* and *B. purificata*, the *O. hupensis* genome shows more expanded and contracted gene families, indicating that there are higher gene additions and loss events in the evolution process to better adapt to the alternation of water and land environments (Fig. 2E). Functional enrichment of the expanded gene families indicated they were mainly intracellularly involved (109 genes, *P*-value = 8.27 × e^−30^), DNA-mediated transposition (92 genes, *P*-value = 7.31 × e^−78^), DNA integration (64 genes, *P*-value = 1.80 × e^−42^), transposase activity (31 genes, *P*-value = 3.79 × e^−24^) and hyalurononglucosaminidase activity biological process (16 genes, *P*-value = 1.16 × e^−07^). DNA-mediated transposition and transposase activity genes play a key role in gene family expansion (Additional file 1: Table S11, S12, Fig. 3A). We also found that there are many significantly expanded gene families in *O. hupensis*, including the protocadherin Fat 4 gene family (33 genes), the F-box protein 20 gene family (21 genes), the histone H2B gene family (15 genes), the gene family of neurotransmitters, olfactory receptors and neuroactive ligand-receptor interaction (14 genes) and tight junction (21genes). Our analysis found that ABC transporters (8 genes, *P*-value = 5.71 × e^−10^), arachidonic acid metabolism (7 genes, *P*-value = 4.36 × e^−8^), linoleic acid metabolism (6 genes, *P*-value = 9.87 × e^−9^), antifolate resistance and (6 genes, *P*-value = 1.54 × e^−8^), ovarian steroidogenesis (6 genes, *P*-value = 2.95 × e^−7^) and other gene families contracted significantly in the snails (Fig. 3B). We also calculated the positive selection genes of the snails using PAML. Totally 281 protein-coding genes under positive selection were identified in *O. hupensis* (FDR < 0.05). KEGG and Gene Ontology (GO) analysis of the positively selected genes showed enrichment in protein kinase activity, protein phosphorylation, catalytic activity, metabolic process, etc. (Fig. 3C, D). Nine protein-coding genes under positive selection were identified in *O. hupensis* (Additional file 1: Table S13). GO and KEGG analysis of the positively selected genes showed enrichment in G-protein-coupled protein receptor activity (GPCR), c1q, F-box protein and protocadherin Fat 4.

**Fig. 4:**
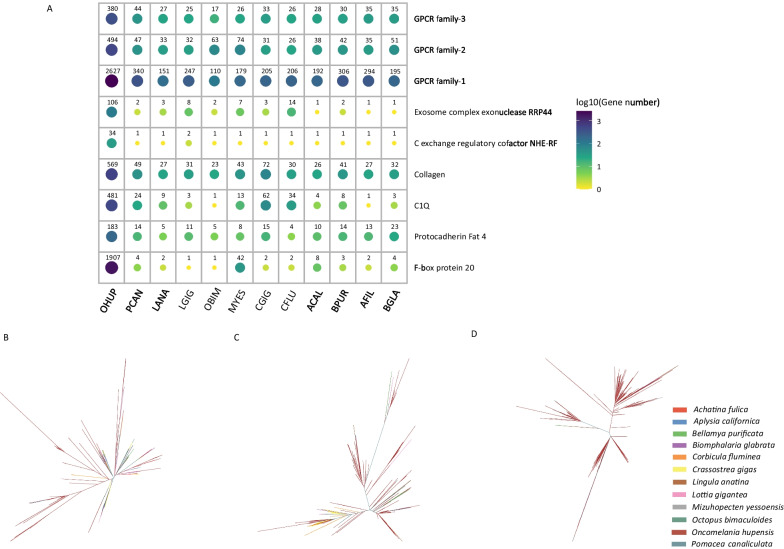
Heatmap map and phylogenetic tree analysis of the identified positively selected genes. **A** Heat map of the identified positively selected genes. **B** Phylogenetic tree analysis of protocadherin Fat 4. **C** Phylogenetic tree analysis of c1q. **D** Phylogenetic tree analysis of F-box protein

By identifying the functional genes of GPCR, protocadherin Fat 4, c1q, and F-box protein in the *O. hupensis* genome and its related species (*B. purificata*, *B. glabrata*, etc.), and drawing a heat map based on the number of genes, the results showed that the number of related functional genes in the *O. hupensis* genome is significantly higher than that of other species. By constructing a phylogenetic tree of these functional genes, the results showed that the relevant functional genes replicate and explode uniquely in the *O. hupensis* genome compared to other species genomes Fig. 4A–D).

## Discussion

*S. japonicum* was transmitted in the Chinese mainland, but now it has been controlled as a result of sustained efforts from the national schistosomiasis control program. *O. hupensis* is the unique intermediate host of *S. japonicum*. The control of the snail is of great significance for consolidating the achievements of schistosomiasis control program, even in the post-elimination stage. However, without knowledge of its genome, the origin of *O. hupensis* and its family Pomatiopsinae were inconclusive. This study solved this conundrum by the generation of a chromosome-scale genome assembly with a scaffold N50 of 75.08 Mb based on third-generation sequencing with the Hi-C technique. The molecular clock evolutionary tree of the single copy genes of the genome data showed that *O. hupensis* is most closely related to *P. canaliculata* and *B. purificata*, which diverged from their common ancestor around 152.70–191.60 million years ago, with the time of divergence between *O. hupensis* and other mollusk species about 354.40 (325.60–423.70) million years ago (Fig. 2D), a fact consistent with previous studies [10, 43]. The successful revelation of the *O. hupensis* genome statutes a foundation for snail genome research that contributes to research of the origin and evolution of the family Pomatiopsinae and its co-evolution with schistosomes.

As the second largest group in nature after insects, mollusks have shown strong in evolutionary adaptability. In contrast to other mollusks, *O. hupensis* has developed a unique amphibious route by adapting to both aquatic and terrestrial habitats. Compared with *P. canaliculata* and *B. purificata*, the *O. hupensis* genome has more expanded and contracted gene families, indicating that there are higher gene additions and loss events in the evolution process to better adapt to the alternation of aquatic and terrestrial environments. Functional enrichment of the expanded gene families indicates that this species has been involved in DNA-mediated transposition, DNA integration and transposase activity, which play key roles in gene family expansion. We also found that there are many significantly expanded gene families in *O. hupensis*, including the protocadherin Fat 4, F-box protein gene family, GPCR and c1q. The former gene family directly affects neuronal synapse development and is also a key regulator of cell growth and animal development, a fact shown to play an important role in both planar cell polarity and cell boundary formation during development [44]. The F-box protein gene family, on the other hand, is one of the most conserved gene families in eukaryotes, which is active at the protein–protein interaction sites and also facilitates programmed cell death [45]. C1q domain-containing proteins (C1qDCs), which are found in a large number of mollusks, such as *Pinctada fucata*, *Zhikong scallop, Chlamys farreri*, *Hyriopsis cumingii*, etc., have been confirmed that these proteins played crucial roles in adaptive and innate immunity in the immune system [46–48]. These expansion genes of *O. hupensis* mainly related to cell development and immune defense mechanism are stronger than that in the other related species, which may have had an impact on the biological functions of *O. hupensis*.

We also found that ABC transporters, arachidonic acid metabolism, linoleic acid metabolism, antifolate resistance and ovarian steroidogenesis gene families contracted significantly in the snails, which may be related to feeding habits, reproductive, environmental adaptation and immune defense behavior [49–51]. In China, the ribbed-shell snail populations and the smooth-shell snail populations are distributed in different ecological environments, and their compatibility with *S. japonicum* is also different. In general, the infection rate of the ribbed-shell snails in marshland and lake region is higher than the smooth-shell snails in mountain and hilly region [12], and this complex compatibility relationship is not only reflected in different large-scale regions, but also within relative small areas. For example, the *O. hupensis* snails in the upper stream of the Miaohe River (Songzi City, Hubei Province, China) are all of the smooth-shell snails that are not infected with *S. japonicum*, while those in the downstream areas of the same river are all ribbed-shell snails accessible for *S. japonicum* infection [52]. The 281 positively selected protein-coding genes showed enrichment in protein kinase activity, protein phosphorylation, catalytic activity and metabolic process. The calcium-responsive transcription factor genes, carbon metabolism genes, anabolic and catabolic genes were under positive selection, which may be related to the adaption to variable nutrition availability and environmental adaptation [53, 54]. The possible relationship between these genes and the regulation of snail shell formation warrants further investigation. The reference genome of *O. hupensis* sheds light on susceptibility mechanisms exhibited by ribbed- or smooth-shell snails, offering novel avenues to explore genetic regulatory approaches for interrupting the transmission of schistosomiasis.

Although this study constructed a chromosome-level genome of *O. hupensis*, there might still be some imperfections such as the PacBio data with an average sub-read length was only 8.02 kbp. There is currently only one male ribbed shell *O. hupensis* was sequenced for genome, no female *O. hupensis* and smooth shell *O. hupensis* was sequenced for genome. So, with the development of genome sequencing technology, more *O. hupensis* genomes will be annotated to study its biology and origin in the near future.

## Conclusions

Using an integrated sequencing strategy combining with technologies of PacBio, Illumina, and Hi-C, we successfully reconstructed the first chromosome-level assembly for *O. hupensis*, predicting a total of 30,604 genes functionally annotated with putative functions clustered into 26,089 gene families. With 1196 single-copy orthologs from *O. hupensis* and other related mollusks, we constructed the phylogenetic relationship of these mollusks and found that *O. hupensis* might have diverged from its common ancestor *P. canaliculata* and *B. purificata* around 152.70–191.60 million years ago. Given the increasing interest in mollusk genomic evolution and the biological importance of *O. hupensis* as the only intermediate host of *S. japonicum*, our genomic and transcriptome data should provide valuable genetic resources for follow-on functional genomics investigations by the research community.

### Supplementary Information

**Additional file 1: Table S1.** General statistics for *O. hupensis* by Hi-C assisted assembly. **Table S2.** Hi-C assisted assembly for *O. hupensis* genome. **Table S3.** Information statistics for *O. hupensis* genome assembly. **Table S4.** BUSCO results for *O. hupensis* genome. **Table S5.** Summary of mapping statistics. **Table S6.** General statistics of gene prediction. **Table S7.** Statistics of repeat sequence annotated by different software. **Table S8.** Statistics of repeat sequence classification. **Table S9.** Statistics of non-coding RNA annotation. **Table S10.** Gene family clustering. **Table S11.** Significantly expanded gene family (*P* < 0.05) GO enrichment of *O. hupensis*. **Table S12.** Protein-coding genes under KEGG positive selection in *O. hupensis* (FDR < 0.05) (partly). **Table S13.** Gene number of the positive selection in *O. hupensis* and other species.

## Data Availability

The whole-genome assembly of *O. hupensis* was submitted to NCBI under PRJNA1033027 and JBAHVR000000000.

## Abbreviations

CDS: Coding sequence
GPCR: G protein-coupled protein receptor activity
GO: Gene ontology
KEGG: Kyoto Encyclopedia of Genes and Genomes
DNA: Deoxyribo nucleic acid
Hi-C: High-throughput chromosome conformation capture
PCR: Polymerase chain reaction
SNPs: Single nucleotide polymorphisms
INDELs: Insertion and deletion
Iso-Seq: Isoform-sequencing
MYA: Million years ago
