# Supplementary material for: Chromosome-level genome assembly of Oncomelania hupensis: the intermediate snail host of Schistosoma japonicum
Source: Infect Dis Poverty. 2024 Feb 27;13:19. doi: 10.1186/s40249-024-01187-3 (PMC10898136; doi:10.1186/s40249-024-01187-3)
Supplement: Supplementary file 1 — Additional file 1: Table S1. General statistics for O. hupensis by Hi-C assisted assembly. Table S2. Hi-C assisted assembly for O. hupensis genome. Table S3. Information statistics for O. hupensis genome assembly. Table S4. BUSCO results for O. hupensis genome. Table S5. Summary of mapping statistics. Table S6. General statistics of gene prediction. Table S7. Statistics of repeat sequence annotated by different software. Table S8. Statistics of repeat sequence classification. Table S9. Statistics of non-coding RNA annotation. Table S10. Gene family clustering. Table S11. Significantly expanded gene family (P < 0.05) GO enrichment of O. hupensis. Table S12. Protein-coding genes under KEGG positive selection in O. hupensis (FDR < 0.05) (partly). Table S13. Gene number of the positive selection in O. hupensis and other species. [file 40249_2024_1187_MOESM1_ESM.docx]

Table S1. General statistics for *O. hupensis* by Hi-C assisted assembly

| **Before/after Hi-C assisted assembly** | **Total sequence**  **length (bp)** | **Contig**  **number** | **Contigs**  **N50 (bp)** | **Scaffold number** | **Scaffold**  **N50 (bp)** |
| --- | --- | --- | --- | --- | --- |
| Sketch sequence  Before Hi-C assisted assembly | 1,516,862,804 | 1512 | 1,834,408 | 1510 | 1,834,408 |
| Anchored to the chromosome sequence  After Hi-C assisted assembly | 1,376,901,521 | 1762 | 1,395,498 | 17 | 75,075,133 |

Table S2. Hi-C assisted assembly for *O. hupensis* genome

| **Chr** | **Number of contigs** | **Length of contigs (bp)** | **Length of chr (bp)** |
| --- | --- | --- | --- |
| Chr1 | 187 | 169,791,004 | 169,884,004 |
| Chr2 | 163 | 138,626,477 | 138.707,477 |
| Chr3 | 126 | 97,825,011 | 97,887,012 |
| Chr4 | 103 | 92,989,143 | 93,040,143 |
| Chr5 | 96 | 81,239,905 | 81,286,906 |
| Chr6 | 100 | 81,138,241 | 81,187,741 |
| Chr7 | 91 | 75,030,133 | 75,075,133 |
| Chr8 | 93 | 73,845,206 | 73,891,206 |
| Chr9 | 96 | 72,239,625 | 72,287,125 |
| Chr10 | 111 | 71,358,936 | 71,413,836 |
| Chr11 | 75 | 69,253,666 | 69,290,666 |
| Chr12 | 127 | 62,772,328 | 62,835,328 |
| Chr13 | 88 | 62,014,596 | 62,058,096 |
| Chr14 | 75 | 60,331,177 | 60,368,177 |
| Chr15 | 95 | 59,030,736 | 59,077,736 |
| Chr16 | 74 | 56,891,622 | 56,928,122 |
| Chr17 | 62 | 52,523,815 | 52,554,315 |
| TOTAL | 1762 | 1,376,901,521 | 1,377,773,023 |

**Note**: (1) Length of Contigs: The total length of the contigs that make up the chromosome.

(2) Length of Chr: In order to connect the contigs together and insert 500 'n' between each two contigs.

Table S3. Information statistics for *O. hupensis* genome assembly

| **Name** | **Scaffold length (bp)** | **Scaffold number** | **Contig length (bp)** | **Contig number** |
| --- | --- | --- | --- | --- |
| Max_len | 169,884,004 | - | 6,422,181 | - |
| N10 | 169,884,004 | 1 | 3,160,974 | 35 |
| N20 | 138,707,477 | 2 | 2,538,121 | 88 |
| N30 | 93,040,143 | 4 | 2,025,958 | 153 |
| N40 | 81,286,906 | 5 | 1,679,370 | 232 |
| N50 | 75,075,133 | 7 | 1,346,093 | 329 |
| N60 | 72,287,906 | 9 | 1,067,657 | 449 |
| N70 | 69,290,666 | 11 | 783,773 | 608 |
| N80 | 60,368,177 | 14 | 522,846 | 835 |
| N90 | 56,928,122 | 16 | 316,648 | 1184 |
| Total_length | 1,449,858,197 | 433 | 1,448,986,695 | 2178 |

**Note**: (1) Max_len: The length of the longest scaffold and contig.

(2) N10: The length and number of scaffold and contig bases whose length is greater than or equal to N10

(3) N20: The length and number of scaffold and contig bases whose length is greater than or equal to N20

(4) N30: The length and number of scaffold and contig bases whose length is greater than or equal to N30

(5) N40: The length and number of scaffold and contig bases whose length is greater than or equal to N40

(6) N50: The length and number of scaffold and contig bases whose length is greater than or equal to N50

(7) N60: The length and number of scaffold and contig bases whose length is greater than or equal to N60

(8) N70: The length and number of scaffold and contig bases whose length is greater than or equal to N70

(9) N80: The length and number of scaffold and contig bases whose length is greater than or equal to N80

(10) N90: The length and number of scaffold and contig bases whose length is greater than or equal to N90

(11) Total_length: The total length and number of scaffold and contig bases.

Table S4. Busco results for *O. hupensis* genome

| **Term** | **BUSCO number** | **Percentage (%)** |
| --- | --- | --- |
| Complete BUSCOs | 890 | 91.10 |
| Complete and single-copy BUSCOs | 864 | 88.34 |
| Complete and duplicated BUSCOs | 26 | 2.70 |
| Fragmented BUSCOs | 33 | 3.37 |
| Missing BUSCOs | 55 | 5.62 |
| Total BUSCO groups searched | 978 | 100.00 |

**Note**: (1) Complete BUSCOs: The genes of busco can be matched completely.

(2) Complete and single-copy BUSCOs: A busco can completely match the previous gene.

(3) Complete and duplicated BUSCOs: One busco can completely match multiple genes.

(4) Fragmented BUSCOs: Only part of the sequences could be aligned with the genes in the busco profile.

(5) Missing BUSCO: There is no gene that can match the busco profile.

(6) Total BUSCO groups searched: Total number of genes in busco groups.

Table S5. [Summary of mapping statistics](http://figshare.com/articles/_Summary_of_mapping_statistics_for_the_Twilight_Sanger_reads_/1462486)

| **Mapping rate (%)** | **Average sequencing depth** | **Coverage (%)** | **Coverage**  **(≥ 5X, %)** | **Coverage**  **(≥ 10X, %)** | **Coverage**  **(≥ 20X, %)** |
| --- | --- | --- | --- | --- | --- |
| 99.15 | 101.47 | 99.65 | 99.50 | 99.37 | 99.03 |

**Note**: (1) Mapping rate: Comparison rate of reads comparison contig.

(2) Average sequencing depth: total number of single base depth/total base number;

(3) Coverage: Bases with depth not less than 1/total bases.

(4) Coverage (≥ 5X): Bases with depth not less than5/total bases.

(5) Coverage (≥ 10X): Bases with depth not less than 10/total bases.

(6) Coverage (≥ 20X): Bases with depth not less than 20/total bases.

Table S6. General statistics of gene prediction

| **Gene set** | | **Number** | **Average gene length (bp)** | **Average CDS length (bp)** | **Average exon per length (bp)** | **Average exon length (bp)** | **Average intron length (bp)** |
| --- | --- | --- | --- | --- | --- | --- | --- |
| *De novo* | AUGUSTUS | 27,617 | 20,671.39 | 1,392.62 | 6.47 | 215.36 | 3526.74 |
|  | Genscan | 55,783 | 17,394.94 | 1,209.16 | 5.34 | 226.47 | 3730.00 |
|  | GeneID | 59,855 | 14,666.21 | 906.34 | 3.56 | 254.48 | 5371.79 |
| Homolog | *A. fulica* | 47,380 | 6,270.07 | 733.83 | 2.53 | 290.59 | 3629.64 |
|  | *A. californica* | 23,755 | 10,313.01 | 691.16 | 3.42 | 201.92 | 3971.21 |
|  | *L. gigantea* | 27,303 | 8,189.28 | 679.78 | 3.25 | 209.15 | 3337.32 |
|  | *B. glabrata* | 29,654 | 9,376.53 | 690.90 | 3.29 | 209.77 | 3791.80 |
|  | *M. yessocensis* | 37,831 | 9,036.04 | 741.78 | 3.10 | 239.32 | 3950.45 |
|  | *P. canaliculata* | 31,017 | 14,454.97 | 857.86 | 4.65 | 184.33 | 3721.19 |
| trans.orf/RNAseq | | 37,831 | 11,721.81 | 689.90 | 3.48 | 694.93 | 3751.22 |
| trans.orf/ISOseq | | 927 | 24,958.72 | 1,112.62 | 7.07 | 399.73 | 3645.43 |
| BUSCO | | 954 | 15,622.31 | 1,289.52 | 10.33 | 124.88 | 1536.66 |
| MAKER | | 30,604 | 20,007.73 | 1,395.39 | 6.13 | 228.09 | 3629.35 |

Note: Five closely related species were selected, namely,. *A.fulica*, *A. californica*, *L. gigantean*, *B. glabrata*, *P. canaliculata*.

Table S7. Statistics of repeat sequence annotated by different software

| **Type** | **Repeat size (bp)** | **% of genome** |
| --- | --- | --- |
| TRF | 144,749,311 | 9.98 |
| RepeatMasker | 327,465,556 | 22.59 |
| ProteinMask | 229,024,698 | 15.80 |
| *De novo* | 537,873,769 | 37.10 |
| Total | 858,074,929 | 59.18 |

**Note**: (1) TRF: The tandem repeats were found by TRF software.

(2) RepeatMasker: Based on the RepBase library, the transposon elements were annotated by RepeatMasker software.

(3) RepeatProteinMask: Based on the RepBase library, the transposon elements were annotated by RepeatProteinMask software.

(4) *De novo*: The final sequence files obtained by software RepeatModeler and LTR-FINDER were used as the library, and the results were annotated by software RepeatMasker.

(5) Total: The results obtained by the above methods are non redundant after removing the overlapped part between them.

Table S8. Statistics of repeat sequence classification

|  | **RepeatMasker TEs** | | **RepeatProteinMask TEs** | | ***De novo*** | | **Combined TEs** | |
| --- | --- | --- | --- | --- | --- | --- | --- | --- |
|  | Length  (bp) | % in genome | Length  (bp) | % in genome | Length  (bp) | % in genome | Length  (bp) | % in genome |
| DNA | 95,627,456 | 6.60 | 1,248,386 | 0.09 | 68,160,741 | 4.70 | 162,216,143 | 11.19 |
| LINE | 177,973,806 | 12.28 | 162,919,073 | 11.24 | 284,426,245 | 19.62 | 421,039,687 | 29.04 |
| SINE | 5,844,867 | 0.40 | 0 | 0.00 | 4781,102 | 0.33 | 10,606,324 | 0.73 |
| LTR | 66,923,379 | 4.62 | 64,892,999 | 4.48 | 206,897,620 | 14.27 | 276,732,498 | 19.09 |
| Other | 287,277 | 0.02 | 0 | 0.00 | 0 | 0.00 | 287,277 | 0.02 |
| Unknown | 2,471,011 | 0.17 | 0 | 0.00 | 58,879,832 | 4.06 | 61,347,316 | 4.23 |
| Total TEs | 295,110,033 | 20.35 | 229,024,698 | 15.80 | 505,322,712 | 34.85 | 759,489,076 | 52.38 |

Note: (1) RepeatMasker TEs: transposon elements obtained from genome annotation by RepeatMasker software based on RepBase library.

(2) RepeatProteinMask TEs: Based on the RepBase library, transposon elements were annotated by RepeatProteinMask software.

(3) De novo: the repeat sequences obtained by de novo prediction method, [RepeatModeler](http://www.baidu.com/link?url=DO9bbZOlHoR9OoLaIXeChVH2jTxAUwYuAQ_Ew_gSlPF5DyO0spbAyblFhPP2f8uUo-sQxHG2x3S5NxL2haxwXq) and [LTR_Finder](http://www.baidu.com/link?url=nMq_XdoLsdTX1DqbBzHYXQXtIrHZuY8V48KzThjC4ubdmzSOGO1FQa4djM3EvP1gqHtktWK-7W_ygSEXSZlRd2yN7h6zf-wBU-2n36UtuzS) were used as the library, and the repeat sequences in the genome were obtained by software RepeatMasker.

(4) Combined TEs: the results of integrating the above three methods and removing redundancy.

(5) DNA: DNA transposon.

(6) LINE: long and scattered repeats (> 1000 bp).

(7) SINE: short and scattered repeats (about 300 bp).

(8) LTR: long terminal repeat (1.5 kbp–10 kbp).

Table S9. Statistics of non-coding RNA annotation

| **Type** | | **Copy** | **Average length (bp)** | **Total length**  **(bp)** | **‱ of genome** |
| --- | --- | --- | --- | --- | --- |
| miRNA | | 10 | 90.60 | 906 | 0.01 |
| tRNA | | 1,307 | 75.31 | 98,425 | 0.68 |
| rRNA | rRNA | 17 | 441.88 | 7512 | 0.05 |
|  | 18S | 5 | 1,209.00 | 6045 | 0.04 |
|  | 28S | 2 | 150.00 | 300 | 0.00 |
|  | 5.8S | 0 | 0.00 | 0 | 0.00 |
|  | 5S | 10 | 116.70 | 1167 | 0.01 |
|  | 8S | 0 | 0.00 | 0 | 0.00 |
| snRNA | snRNA | 69 | 154.38 | 10,652 | 0.07 |
|  | CD-box | 8 | 220.12 | 1761 | 0.01 |
|  | HACA-box | 0 | 0.00 | 0 | 0.00 |
|  | splicing | 61 | 145.75 | 8891 | 0.06 |
|  | scaRNA | 0 | 0.00 | 0 | 0.00 |

Table S10 gene family clustering.

| Species | Genes number | Unclustered genes | Genes in families | Family number | Unique families | Unique families genes | Common families | Common families genes | Single copy genes | Average genes per family |
| --- | --- | --- | --- | --- | --- | --- | --- | --- | --- | --- |
| *O. hupensis* | 30,604 | 4515 | 26,089 | 12,366 | 707 | 4763 | 4617 | 5584 | 1196 | 2.11 |
| *P. canaliculata* | 21,144 | 2221 | 18,923 | 12,760 | 355 | 1698 | 4617 | 5674 | 1196 | 1.48 |
| *L. anatine* | 27,068 | 3156 | 23,912 | 11,738 | 1,550 | 6211 | 4617 | 7668 | 1196 | 2.04 |
| *L. gigantea* | 23,818 | 4395 | 19,423 | 12,642 | 640 | 3354 | 4617 | 5711 | 1196 | 1.54 |
| *O. bimaculoides* | 15,684 | 2593 | 13,091 | 9,539 | 299 | 1613 | 4617 | 5733 | 1196 | 1.37 |
| *M. yessoensis* | 24,532 | 3741 | 20,791 | 13,709 | 814 | 2891 | 4617 | 5870 | 1196 | 1.52 |
| *C. gigas* | 31,371 | 3336 | 28,035 | 13,868 | 1,445 | 7326 | 4617 | 6087 | 1196 | 2.02 |
| *C. fluminea* | 27,890 | 4528 | 23,362 | 12,153 | 1,432 | 5317 | 4617 | 6515 | 1196 | 1.92 |
| *A. californica* | 19,424 | 4423 | 15,001 | 11,830 | 312 | 1134 | 4617 | 5345 | 1196 | 1.27 |
| *B. purificata* | 21,646 | 4100 | 17,546 | 12,972 | 410 | 1453 | 4617 | 5589 | 1196 | 1.35 |
| *A. fulica* | 23,726 | 4348 | 19,378 | 12,565 | 410 | 1506 | 4617 | 6655 | 1196 | 1.54 |
| *B. glabrata* | 25,330 | 5743 | 19,587 | 13,381 | 825 | 2918 | 4617 | 5930 | 1196 | 1.46 |

Table S11 Significantly expanded gene family (p < 0.05) GO enrichment of *O. hupensis*

| ID | Description | GeneRatio | BgRatio | p value | p.adjust | q value |
| --- | --- | --- | --- | --- | --- | --- |
| GO:0005622 | intracellular | 109/978 | 392/11325 | 8.27144409448123e-30 | 3.58429244094187e-28 | 3.25053241607684e-28 |
| GO:0007264 | small GTPase mediated signal transduction | 50/978 | 112/11325 | 2.25995561213489e-24 | 7.34485573943838e-23 | 6.66092180418704e-23 |
| GO:0004803 | transposase activity | 31/978 | 43/11325 | 3.7911934202533e-24 | 9.85710289265858e-23 | 8.93923501196568e-23 |
| GO:0007156 | homophilic cell adhesion via plasma membrane adhesion molecules | 32/978 | 92/11325 | 2.01901759424288e-12 | 3.84197136713471e-11 | 3.48421694833269e-11 |
| GO:0003924 | GTPase activity | 56/978 | 238/11325 | 2.06875381307253e-12 | 3.84197136713471e-11 | 3.48421694833269e-11 |
| GO:0016500 | protein-hormone receptor activity | 11/978 | 16/11325 | 5.49816391162875e-09 | 8.93451635639672e-08 | 8.10255734345289e-08 |
| GO:0004415 | hyalurononglucosaminidase activity | 8/978 | 10/11325 | 1.15724961482156e-07 | 1.67158277696448e-06 | 1.51592932000017e-06 |
| GO:0000786 | nucleosome | 16/978 | 42/11325 | 1.63678580121345e-07 | 2.12782154157749e-06 | 1.92968431300954e-06 |
| GO:0005525 | GTP binding | 58/978 | 344/11325 | 4.80634540239363e-07 | 5.68022638464702e-06 | 5.15129842170418e-06 |
| GO:0008408 | 3'-5' exonuclease activity | 11/978 | 23/11325 | 9.6388419934502e-07 | 1.04420788262377e-05 | 9.46973950233704e-06 |
| GO:0003887 | DNA-directed DNA polymerase activity | 10/978 | 23/11325 | 8.86947017054711e-06 | 8.86947017054711e-05 | 8.04356808988888e-05 |
| GO:0006915 | apoptotic process | 13/978 | 41/11325 | 2.444774606513e-05 | 0.000227014784890493 | 0.000205875756337937 |
| GO:0004523 | RNA-DNA hybrid ribonuclease activity | 8/978 | 19/11325 | 9.56774047518372e-05 | 0.000829204174515922 | 0.000751990830330229 |
| GO:0003713 | transcription coactivator activity | 6/978 | 13/11325 | 0.000412067289901116 | 0.00334804673044656 | 0.00303628529400822 |
| GO:0006260 | DNA replication | 10/978 | 43/11325 | 0.00302647902327338 | 0.0231436631191493 | 0.0209885851768804 |
| GO:0000166 | nucleotide binding | 11/978 | 53/11325 | 0.00490263957897173 | 0.0354079525147958 | 0.0321108557219201 |
| GO:0004842 | ubiquitin-protein transferase activity | 12/978 | 84/11325 | 0.0563221929499361 | 0.385362372815352 | 0.349478427168579 |
| GO:0016887 | ATPase activity | 12/978 | 105/11325 | 0.193852473705952 | 0.999999999996881 | 0.906882591090289 |
| GO:0005509 | calcium ion binding | 37/978 | 377/11325 | 0.227685286890654 | 0.999999999996881 | 0.906882591090289 |

Table S12 Protein-coding genes under KEGG positive selection in *O. hupensis* (FDR < 0.05) (partly).

| Gene | Pvalue | FDR | Site num |
| --- | --- | --- | --- |
| Ohu012645 | 0.000467560613284457 | 4.333489e-03 | 2 |
| Ohu010150 | 0.00225176862677773 | 1.458532e-02 | 1 |
| Ohu011796 | 0.00407205405497524 | 2.266618e-02 | 3 |
| Ohu007612 | 0.00694489970606826 | 3.326549e-02 | 2 |
| Ohu027101 | 0.00253018587300885 | 1.576181e-02 | 30 |
| Ohu000972 | 5.08377848518204e-06 | 1.093492e-04 | 51 |
| Ohu001399 | 0.00105988423286696 | 8.109181e-03 | 12 |
| Ohu028938 | 0.0108836500269005 | 4.544821e-02 | 7 |
| Ohu000836 | 0.000540643888680026 | 4.754489e-03 | 18 |
| Ohu002330 | 0.000444939167345171 | 4.191989e-03 | 17 |
| Ohu015157 | 0.0105985508046665 | 4.442040e-02 | 7 |
| Ohu001643 | 5.37637450581663e-06 | 1.135012e-04 | 27 |
| Ohu002540 | 0.00044299182411589 | 4.191989e-03 | 18 |
| Ohu002397 | 0.00593901057254426 | 2.995784e-02 | 4 |
| Ohu012888 | 0.000566362641237439 | 4.891314e-03 | 22 |
| Ohu012807 | 0.0120750388414931 | 4.933887e-02 | 8 |
| Ohu025384 | 0.00195185762768224 | 1.348556e-02 | 16 |
| Ohu001991 | 4.14689799166368e-08 | 1.818255e-06 | 37 |
| Ohu015388 | 2.2306052005483e-09 | 1.412717e-07 | 12 |
| Ohu014501 | 6.24199687919713e-05 | 9.122919e-04 | 51 |
| Ohu010675 | 0.00757283257235197 | 3.495153e-02 | 13 |

Table S13 Gene number of the positive selection in *O. hupensis* and other species.

| Species  function | *Oncomelania hupensis* | *Pomacea canaliculata* | *Lingula anatina* | *Lottia gigantea* | *Octopus bimaculoides* | *Mizuhopecten yessoensis* | *Crassostrea gigas* | *Corbicula fluminea* | *Aplysia californica* | *Bellamya purificata* | *Achatina fulica* | *Biomphalaria glabrata* |
| --- | --- | --- | --- | --- | --- | --- | --- | --- | --- | --- | --- | --- |
| F-box protein 20 | 1,907 | 4 | 2 | 1 | 1 | 42 | 2 | 2 | 8 | 3 | 2 | 4 |
| protocadherin Fat 4 | 183 | 14 | 5 | 11 | 5 | 8 | 15 | 4 | 10 | 14 | 13 | 23 |
| C1Q | 481 | 24 | 9 | 3 | 1 | 13 | 62 | 34 | 4 | 8 | 1 | 3 |
| Collagen | 569 | 49 | 27 | 31 | 23 | 43 | 72 | 30 | 26 | 41 | 27 | 32 |
| C exchange regulatory cofactor NHE-RF | 34 | 1 | 1 | 2 | 1 | 1 | 1 | 1 | 1 | 1 | 1 | 1 |
| exosome complex exonuclease RRP44 | 106 | 2 | 3 | 8 | 2 | 7 | 3 | 14 | 1 | 2 | 1 | 1 |
| GPCR family-1 | 2,627 | 340 | 151 | 247 | 110 | 179 | 205 | 206 | 192 | 306 | 294 | 195 |
| GPCR family-2 | 494 | 47 | 33 | 32 | 63 | 74 | 31 | 26 | 38 | 42 | 35 | 51 |
| GPCR family-3 | 380 | 44 | 27 | 25 | 17 | 26 | 33 | 26 | 28 | 30 | 35 | 35 |
